# Breast density is strongly associated with multiparametric magnetic resonance imaging biomarkers and pro-tumorigenic proteins in situ

**DOI:** 10.1038/s41416-022-01976-3

**Published:** 2022-09-22

**Authors:** Peter Lundberg, Mikael F. Forsgren, Jens Tellman, Johan Kihlberg, Anna Rzepecka, Charlotta Dabrosin

**Affiliations:** 1 Department of Radiation Physics and Department of Medical and Health Sciences, Linköping University, Linköping, Sweden; 2 Center for Medical Image Science and Visualization (CMIV), Linköping University, Linköping, Sweden; 3 AMRA Medical AB, Linköping, Sweden; 4 Department of Radiology and Department of Medical and Health Sciences, Linköping University, Linköping, Sweden; 5 Department of Oncology and Department of Biomedical and Clinical Sciences, Linköping University, Linköping, Sweden

**Keywords:** Translational research, Molecular medicine

## Abstract

**Background:**

High mammographic density is an independent risk factor for breast cancer by poorly understood molecular mechanisms. Women with dense breasts often undergo conventional magnetic resonance imaging (MRI) despite its limited specificity, which may be increased by diffusion-weighted imaging (DWI) with apparent diffusion coefficient (ADC) and contrast. How these modalities are affected by breast density per se and their association with the local microenvironment are undetermined.

**Methods:**

Healthy postmenopausal women attending mammography screen with extremely dense or entirely fatty breasts underwent multiparametric MRI for analyses of lean tissue fraction (LTF), ADC and perfusion dynamics. Microdialysis was used for extracellular proteomics in situ.

**Results:**

Significantly increased LTF and ADC and delayed perfusion were detected in dense breasts. In total, 270 proteins were quantified, whereof 124 related to inflammation, angiogenesis, and cellular growth were significantly upregulated in dense breasts. Most of these correlated significantly with LTF, ADC and the perfusion data.

**Conclusions:**

ADC and perfusion characteristics depend on breast density, which should be considered during the implementation of thresholds for malignant lesions. Dense and nondense breasts are two essentially different biological entities, with a pro-tumorigenic microenvironment in dense breasts. Our data reveal several novel pathways that may be explored for breast cancer prevention strategies.

## Introduction

Mammographic breast density is a strong independent risk factor for breast cancer [1, 2]. Despite this known association, the pathophysiological mechanisms underlying this increased risk are not fully understood. Dense breast tissue, which appears white or light grey on mammograms, contains a higher amount of stroma, including collagen, and less fat tissue [3, 4]. Normal breast tissue contains 1–6% epithelial cells [1, 5, 6], which contributes to its density. The amount of epithelial cells has, in some studies, been reported to be increased in dense breasts, whereas others have failed to show any difference depending on breast density [3–5, 7, 8]. In postmenopausal women, however, collagen, but not epithelial or glandular area, is associated with mammographic density [9]. We recently showed that dense breast tissue, measured using lean tissue fraction (LTF) as an accurate global measurement of breast density, in postmenopausal women exhibits a pro-inflammatory, pro-angiogenic microenvironment similar to that found in human breast cancer [10, 11].

The sensitivity of detecting malignancies is decreased in mammographically dense breasts, with a reported drop from 80% in nondense breasts to 30% in dense breasts [12, 13]. Due to these known diagnostic challenges, women with dense breasts are more likely to undergo additional diagnostic procedures such as conventional magnetic resonance imaging (MRI). MRI has significantly higher sensitivity compared with mammography, but its limited specificity can lead to unnecessary biopsies. Therefore, complementary techniques such as diffusion-weighted imaging (DWI) may reduce unnecessary breast biopsies [14]. DWI can be used to measure the mobility of water molecules diffusing in tissue, which can be quantified through the apparent diffusion coefficient (ADC) [15]. ADC has primarily been used to discriminate between benign and malignant lesions and thereby reduce the number of unnecessary biopsies [16]. In general, malignant lesions exhibit lower ADCs, which may be the result of increased cellularity in cancers. Extracellular oedema is also suggested to increase ADC, but the underlying mechanisms are not yet fully understood [17]. Recently, an optimised threshold of ADC was suggested to reduce the number of unnecessary biopsies of suspicious breast lesions [18]. However, it is still unclear whether patient characteristics, including stromal characteristics surrounding lesions, may directly or indirectly affect ADC measurements. A few previous studies have determined the ADC of normal breast and its relation fibroglandular and adipose tissue and to the menstrual cycle [19–22]. Moreover, the use of DWI (or ADC) for screening purposes may be useful considering the limited value of conventional mammography in women with dense breasts, at least for a selected patient population [23].

The addition of a contrast agent can increase the specificity of MRI [24]. Using dynamic contrast-enhanced (DCE) technique, or perfusion MRI, images are acquired during the passage of the contrast agent through tissue, thus allowing evaluation of the integrity of the vasculature in tissue [25]. DCE-MRI parameters are related to tumour angiogenesis and may serve as useful functional measures of blood flow and vascular permeability in the evaluation of anti-angiogenic therapies [26].

There is clearly a need for improved identification of determinants of breast density to provide insights into the aetiology of breast cancer. With this knowledge in hand, improved prevention strategies can be developed. There is also a need for a biological understanding of factors that affect ADC and perfusion dynamics in normal breast tissue, which may indirectly affect the identification of pathological lesions.

The aim of this study was to quantify selected imaging biomarkers that specifically correlate with regional microstructural tissue properties in healthy postmenopausal women with dense or nondense breasts. In addition, we correlated these properties with an extended range of extracellular proteins from breast tissue in situ to reveal possible biological mechanisms underlying alterations in imaging biomarkers.

## Materials and methods

### Participants

This study was performed in accordance with the Declaration of Helsinki and was approved by the regional ethical review board of Linköping. All women gave informed written consent. A total of 44 postmenopausal women (55 years of age or older) were consecutively recruited from the screening mammography programme at Linköping University Hospital during 2014–2015. The recruitment procedure is depicted in a STROBE diagram in Supplementary Materials. Regular mammograms were assessed by one experienced observer (AR) according to the Breast Imaging Reporting and Data System (BI-RADS) density scale [27], and breast densities were categorised as either BI-RADS A (entirely fatty nondense breasts) or BI-RADS D (extremely dense). As this was the first explorative study of its kind, we chose to include the BI-RADS A and D groups only to be able to reveal if any differences at all would be possible to detect using this set-up.

No previous breast cancer or benign breast disease, current use of hormone replacement therapy or anti-oestrogen therapies including selective oestrogen receptor modulators or degraders, any clotting or metabolic disorder, or current use of non-steroidal anti-inflammatory drugs were allowed.

### Magnetic resonance imaging

Participants were imaged using a 1.5 T Achieva MR scanner (Philips Healthcare, Best, Netherlands) with a dual-breast seven-element breast coil. LTF was computed as the ratio of lean tissue volume to total volume as previously described [11]. LTF was based on water- and fat-separated MR images computed from axial 3D 6-echo turbo field echo MRI images acquired on a, anterior–posterior frequency encoding, first TE at 2.3 ms and DTE of 2.3 ms, TR 15.4 ms, flip angle 10°, 300 × 300 × 150 mm^3^ field of view (FOV), 200 × 200 scan matrix and 3-mm slice thickness.

An echo-planar (EPI) single shot DWI was performed with an EPI factor of 61, TE 83.1 ms, TR 11282.8 ms, three averages, 160 × 157 scan matrix, 3-mm slice thickness, no slice distance, in-plane resolution of 2.0 × 2.0 mm^2^ (reconstructed into 1.4 × 1.4 mm^2^) and b = 100, 400 and 800 s/mm^2^ using SPectral Attenuated Inversion Recovery (SPAIR) for fat suppression. ADC maps were computed using manufacturer-provided MR scanner software.

Perfusion data were acquired with a 3D spoiled turbo gradient echo sequence (eTHRIVE), TE 3.4 ms, TR 6.9 ms, Flip Angle 10°, 332 × 339 scan matrix, 2-mm slice thickness, with 1 mm overlap, in-plane resolution of 1.0 × 1.0 mm^2^ (reconstructed into 1.0 × 1.0 mm^2^) using SPAIR for fat suppression. Following one baseline image acquisition, an intravenous bolus injection of 15 mL Dotarem^®^ (Gadoteric acid, Guerbet, Villepinte, France) was administered at a rate of 1 mL/using a power injector (Medrad Spectris Solaris, Pittsburgh, PA, USA) followed by a 30 mL saline flush. Post-contrast images were typically acquired 110, 180, 240, 300 and 360 s following the injection.

Co-localisation and analysis of LTF, DWI and perfusion dynamics as imaging biomarkers were performed using Matlab (Version 2020b, The Mathworks Inc, Natick, Massachusetts, USA). Co-localisation was performed by manually placing a virtual “MR-spectroscopy-sized” 20 × 20 × 20 mm^3^ volume of interest (VOI) in the upper lateral quadrant of the left breast within the glandular tissue in the eTHRIVE pre-contrast agent injection image volume of each subject. Each placed VOI was matched in the corresponding LTF and DWI volumes through an automated process. Volumetric and positional information from the metadata of the images was used to find the corresponding voxel coordinates of the VOI in the LTF and DWI volumes. Voxels partially outside the VOI were excluded.

Perfusion data were analysed as follows; the mean intensity values within each selected VOI were normalised to their corresponding baseline values. This was applied to all acquisitions in the perfusion series. The normalised data points were fitted in Matlab, using a non-linear least-squares algorithm, to a phenomenological model that describes the time-dependent relative enhancement (RE) and rise time of the relative enhancement (τ or tau)

1 $$RE\left( t \right) = A\left( {1 - Be^{ - \frac{t}{\tau }}} \right),$$

where A is the mean of the last two timepoints and B is a constant. The perfusion parameter area under the curve (AUC) was determined as the integral of the fitted curve with the time of contrast agent injection as the starting point (c.100 s after baseline measurement) and 240 s as the standardised endpoint of the integration. The perfusion parameter τ was estimated from the fit of the perfusion data points.

Background parenchymal enhancement (BPE) was quantitated by one experienced observer (AR) and categorised from 1 to 4 representing minimal, mild, moderate and marked.

### Microdialysis procedure

Microdialysis was performed in the upper lateral quadrant of the left breast in all women at the same location as used for MR measurements. Prior to insertion of the microdialysis catheter, 0.5 mL lidocaine (10 mg*/*mL) was administrated intracutaneously. During insertion, the microdialysis catheter was directed toward the nipple as previously described [28–37]. Microdialysis catheters (71*/*M Dialysis AB, Stockholm, Sweden) consist of a tubular dialysis membrane (length 20 mm, diameter 0.52 mm, 100,000 atomic mass cut-off) glued to the end of a double-lumen tube (80 mm length × 0.8 mm diameter) were inserted via a splitable introducer (M Dialysis AB) connected to a microinfusion pump (M Dialysis AB) and perfused with NaCl 154 mmol*/*L and hydroxyethyl starch 60 g/L (Voluven^®^, Fresenius Kabi, Uppsala, Sweden) at a rate of 0.5 µL/min. After a 60-min equilibration period, the outgoing perfusate was stored at −70 °C for subsequent analysis. After the microdialysis investigation, an unguided breast biopsy was obtained from the upper lateral quadrant of the left breast (MAX-CORE, gauge 14, BARD Medical, Covington, GA, USA) and fixed in 4% paraformaldehyde.

### Protein quantification

Samples were analysed using a multiplex proximity extension assay (Olink Bioscience, Uppsala, Sweden) with Inflammation, Cardiovascular II, Cardiovascular III, and Cardio Metabolic panels. In brief, 1 μL sample was incubated in the presence of proximity antibody pairs tagged with DNA reporter molecules. Once the pair of antibodies bound to their corresponding antigens, the respective DNA tails formed an amplicon by proximity extension, which was quantified by high-throughput real-time PCR (BioMark™ HD System, Fluidigm Corporation). The generated fluorescent signal is directly correlated with protein abundance. The output from the Proseek Multiplex protocol was quantitation cycles produced by the BioMark’s Real-Time PCR Software. To minimise variation within and between runs, data were normalised using both an internal control (extension control) and an interplate control and then transformed using a pre-determined correction factor. Pre-processed data were provided as the arbitrary unit normalised protein expression on a log_2_ scale and were then linearised using the formula 2^NPX^. A high normalised protein expression value corresponds to a high protein concentration. However, the value is a relative quantification, meaning that no comparison of absolute levels between different proteins can be made.

### Statistical analyses

Data were analysed using unpaired Mann–Whitney *U* tests and Spearman’s correlation tests. A *P* < 0.05 was considered as statistically significant. Proteomic data were analysed using Benjamini–Hochberg correction to correct for the false discovery rate (FDR), which was set to 5%. Statistical analyses were performed with Prism 7.0 (GraphPad software).

In the supplementary data localised ADC measurements, LTF, IQR, Kurtosis and Excess Kurtosis, mean and median were calculated using unpaired *t* tests at the 95% level.

## Results

After re-evaluation of mammograms, one woman in each group were excluded due to mis-categorisation. Three women (two in the dense group and one in the nondense group) were only included in the MR examinations and did not participate in the microdialysis investigation, and two women did not complete the MR examinations but were included in the microdialysis investigation. Moreover, some data were partially corrupted in the storage process in the picture archiving system and could not be recovered. Therefore, 34 women were included in the correlational analyses of LTF, perfusion dynamics, and protein levels, and 36 women were included in the correlational analyses of ADC and protein levels, Supplementary Table S1. There were no differences in age between the groups; median age (range) in BI-RADS A was 66 years (55–73), *n* = 21 vs. 64 years (55–74) in BI-RADS D, *n* = 20 or of BMI (median (range)), which was 25 (19–30) in BI-RADS A vs. 24 (19–32) in BI-RADS D.

**Fig. 1:**
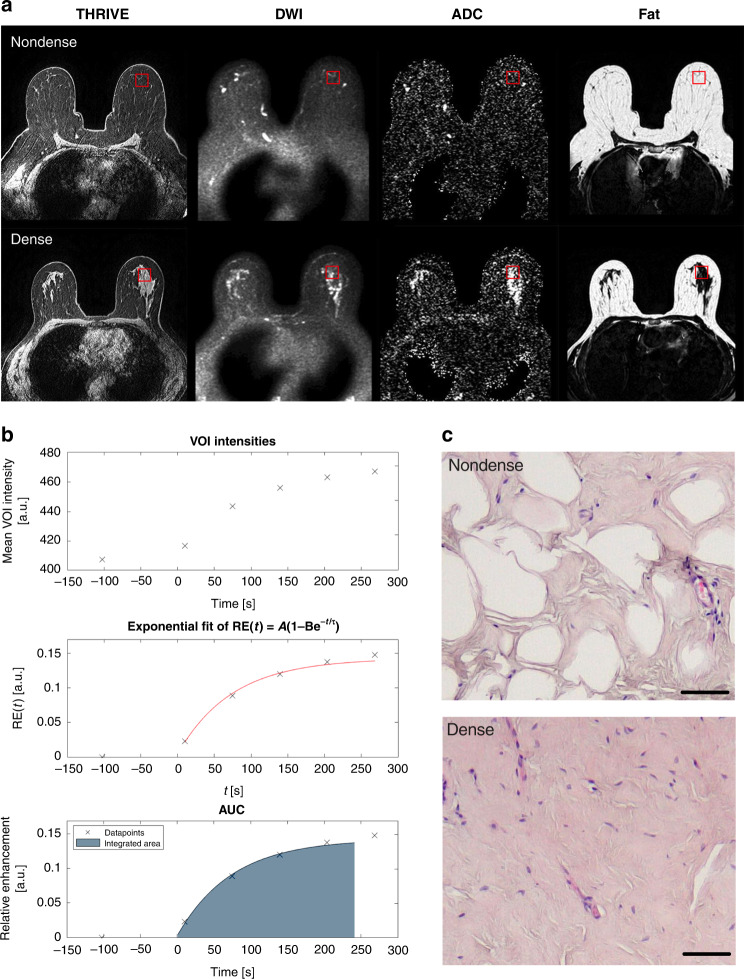
Dense and nondense breasts, THRIVE, ADC and fat measurements. **a** Superimposed is the VOI (in red) used for data extraction of included imaging voxels. Images from left to right; THRIVE pre-contrast agent injection used for perfusion assessment, DWI, ADC maps and fat channel in the water–fat-separated Dixon images for calculating LTF. Top panels depict nondense breasts and bottom panels depict dense breasts. **b** Typical example of a curve fit of THRIVE perfusion data. Initial baseline followed by injection of contrast agent at time 0. Normalised curve fitting provided the time constant (tau) for contrast agent uptake in the VOI as well as the integrated AUC (in grey). **c** Representative hematoxylin and eosin-stained sections from nondense and dense breast tissue. Bars = 50 µm.

Figure 1a shows typical examples of multimodal MR images for nondense and dense breasts. THRIVE pre-contrast agent injection images used for perfusion assessment, ADC maps, DWI and the fat channel (from water–fat-separated Dixon images) used for calculating the LTF are also shown.

VOIs were positioned in a localised region of breast images as close as possible to the subsequent microdialysis and biopsy sites. Figure 1b shows a typical perfusion curve and the curve fit used to extract dynamic characteristics from the data. Figure 1c depicts typical histological characteristics of dense and nondense breast tissue at the site of a regional VOI.

### Increased LTF, ADC and time to contrast peak in dense breast tissue

Diffusion is typically restricted in tissue, with the level of restriction depending on the microstructure and diffusional properties of the endothelium. The level of restriction can be described as the ADC. The physiological details of restricted diffusion and how they affect such measurements are described in Supplementary Figs. S1–3.

**Fig. 2:**
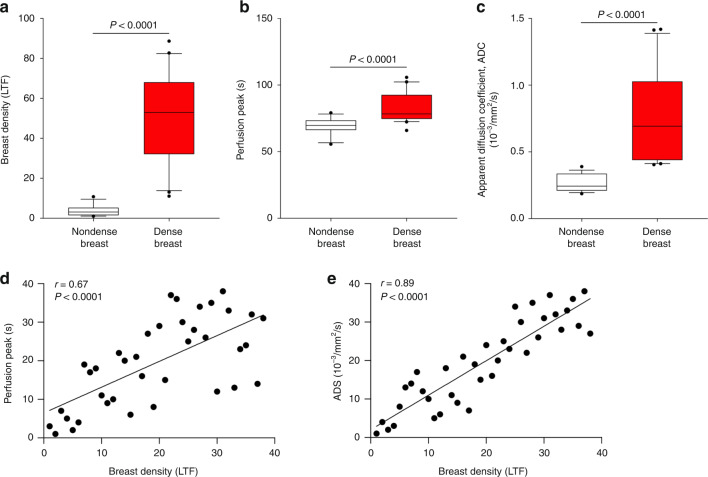
MRI quantification of LTF, time to contrast peak (perfusion tau), and ADC in nondense and dense breast tissue. Postmenopausal women attending a regular mammography screening programme and categorised as having dense or nondense breasts underwent MRI for research purposes. LTF, perfusion tau and ADC were quantified as described in “Materials and methods”. **a** LTF; nondense breasts *n* = 16, dense breasts *n* = 20. **b** Perfusion peak (tau); nondense breasts *n* = 17, dense breasts *n* = 20. **c** ADC; nondense breasts *n* = 17, dense breasts *n* = 20. **d** Correlation between LTF and perfusion tau. **e** Correlation between LTF and ADC. Box plots represent median and 10–90th percentiles.

Corroborating previous measures of LTF calculated in total breast volume [11], LTF in the regional VOI exhibited highly significant differences between dense and nondense breasts (Fig. 2a). The localised LTF in nondense breasts exhibited very little variation, whereas the variation was much larger in the VOIs of dense breasts (Supplementary Fig. S4).

In the same VOI, the characteristic time constant, or tau, was significantly longer in dense breasts than in nondense breasts (Fig. 2b and Supplementary Fig. S5). Corresponding values were observed for the AUC (Supplementary Fig. S5). ADC was significantly increased in dense breast tissue (Fig. 2c and Supplementary Fig. S3). Measures of tissue heterogeneity were also determined using the interquartile range (Supplementary Fig. S3). Corresponding measurements of excess kurtosis in the VOIs indicated more impediments and greater complexity of the microstructure in nondense breast tissue. Moreover, LTF exhibited highly significant positive correlations with perfusion tau and ADC (Fig. 2d, e).

The perfusion parameter τ was estimated from the fit of the perfusion data points as depicted in Supplementary Fig. S6.

There was no difference in BPE between nondense and dense breasts and only a few proteins correlated significantly with the BPE categories, Supplementary Fig. S7

### Proteomic analysis of dense vs. nondense breast tissue

**Fig. 3:**
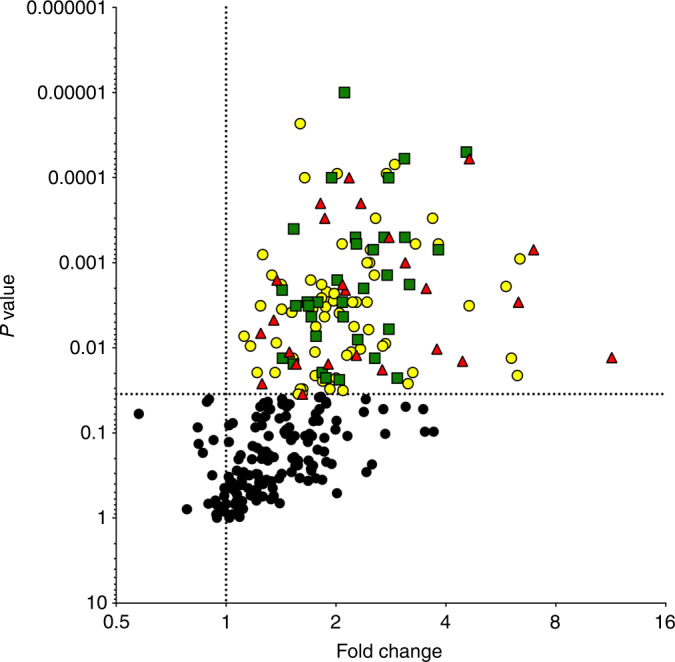
Molecular characterisation of the extracellular microenvironment in vivo in dense breast tissue compared with nondense breast tissue. Microdialysis was performed in postmenopausal women, and the dialysate was analysed as described in “Materials and methods”. Volcano plots illustrate the log_10_ statistical significance (FDR-adjusted *P* value) in relation to the log_2_ fold change of 357 proteins, of which 270 were above the limit of detection. Fold change was calculated from the median value for dense breast tissue (*n* = 20) divided by that for nondense breast tissue (*n* = 20). FDR-adjusted *P* values were set at <0.035. Yellow circles represent inflammatory proteins, red triangles represent angiogenic proteins, and green squares represent proteins related to growth and/or metabolism.

As the microdialysis catheter was inserted in the VOI, local measures obtained by MRI may be more representative of the microenvironment as compared with a previously used global measure [11]. A total of 368 proteins were quantified in the microdialysates using four panels of 92 proteins each. Ten proteins were included in two or more panels; thus, 357 individual proteins were analysed (Supplementary Table S2). Of these 357 proteins, 270 proteins were above the limit of detection in ≥50% of the samples and included in further analyses. These 270 proteins were compared between dense and nondense breast tissues, and fold change was calculated by comparing the median value for dense breast tissue divided by that for nondense breast tissue. After FDR correction, 124 proteins were significantly upregulated in dense breast tissue (*P* < 0.035, Fig. 3). These 124 proteins were divided into three groups: one group of proteins mainly involved in the regulation of inflammation (marked in yellow in Fig. 3), one group mainly related to growth factors/metabolism (marked in green in Fig. 3), and one group mainly involved in angiogenesis (marked in red in Fig. 3).

### Significant correlations between inflammatory proteins and LTF, ADC and perfusion tau

**Fig. 4:**
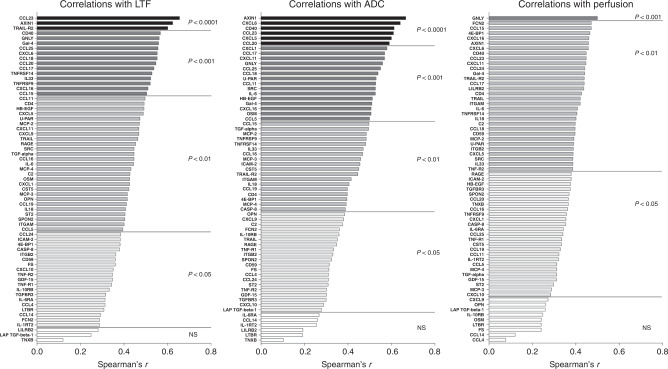
Correlations between inflammatory proteins and LTF, ADC and perfusion tau. A total of 44 postmenopausal women were invited to undergo MRI and microdialysis for the collection of extracellular proteins as described in “Materials and methods”. Complete data for correlation analyses of all parameters were *n* = 35 for LTF and perfusion tau and *n* = 36 for ADC. Bars represent Spearman’s rank correlation coefficients. Coloured bars indicate statistical significance, and white bars indicate ns, not significant. No negative correlations were detected.

A total of 65 proteins involved in the regulation of inflammation were upregulated in dense breast tissue. As shown in Fig. 4, most of these proteins correlated significantly with LTF (62 proteins), ADC (59 proteins) and perfusion tau (56 proteins).

### Significant correlations between growth factors/metabolic proteins and LTF, ADC and perfusion tau

**Fig. 5:**
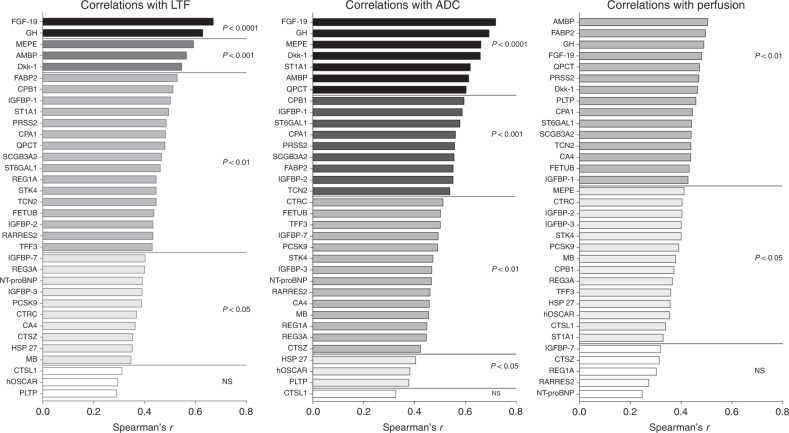
Correlations between proteins related to growth and/or metabolism and LTF, ADC and perfusion tau. A total of 44 postmenopausal women were invited to undergo MRI and microdialysis for the collection of extracellular proteins as described in “Materials and methods”. Complete data for correlation analyses of all parameters were *n* = 35 for LTF and perfusion tau and *n* = 36 for ADC. Bars represent Spearman’s rank correlation coefficient. Coloured bars indicate statistical significance, and white bars indicate ns, not significant. No negative correlations were detected.

A total of 34 proteins mainly involved in growth and/or metabolism were upregulated in dense breast tissue. As shown in Fig. 5, most of these proteins correlated significantly with LTF (31 proteins), ADC (33 proteins) and perfusion tau (29 proteins).

### Significant correlations between angiogenic proteins and LTF, ADC and perfusion tau

**Fig. 6:**
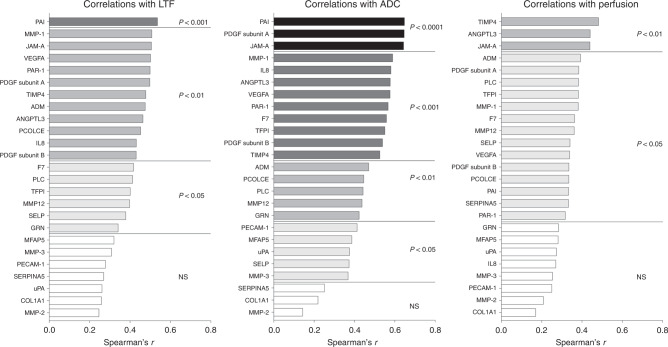
Correlations between angiogenic proteins and LTF, ADC and perfusion tau. A total of 44 postmenopausal women were invited to undergo MRI and microdialysis for the collection of extracellular proteins as described in “Materials and methods”. Complete data for correlation analyses of all parameters were *n* = 35 for LTF and perfusion tau and *n* = 36 for ADC. Bars represent Spearman’s rank correlation coefficient. Coloured bars indicate statistical significance, and white bars indicate ns, not significant. No negative correlations were detected.

A total of 25 proteins mainly involved in angiogenesis were upregulated in dense breast tissue. As shown in Fig. 6, most of these proteins correlated significantly with LTF (18 proteins), ADC (22 proteins) and perfusion tau (17 proteins).

## Discussion

We provide for the first time, to our knowledge, a comprehensive regional multimodal image and molecular characterisation of dense and nondense breast tissue in postmenopausal women. Our study shows that dense breast tissue exhibited a range of different properties linked to tissue microstructure, including increased LTF, slower perfusion dynamics, and increased and more heterogeneous ADC as compared with nondense breast tissue. Additionally, there were significant positive correlations between these parameters. Importantly, proteomic analysis of the extracellular microenvironment revealed significantly increased levels of proteins related to inflammation, cell growth/metabolism, and angiogenesis in dense breasts. Most of these proteins also correlated significantly with LTF, ADC and perfusion dynamics, thus linking molecular differences to tissue microstructure.

The women in our study were initially selected based on BI-RADS classification of the whole breast on regular mammograms, and our data confirmed a significant difference in breast density as assessed by LTF measured locally in the upper lateral quadrant of the left breast. LTF, ADC and perfusion dynamics were objectively and continuously assessed using high-resolution MRI in the same specific tissue region as our molecular characterisation, which strengthens the results of correlation analyses between imaging modalities and protein levels.

Conventional breast MRI provides increased sensitivity in detecting breast cancer compared with mammography, but many benign lesions may nevertheless not be distinguished from malignancies, resulting in unnecessary biopsies. Because of this, the use of ADC has been proposed to decrease false positives and thereby reduce the number of unnecessary biopsies [18]. Before this can be implemented, however, the background ADC in breast tissue must be determined. We show here that ADC depends on tissue density, with a significant decrease in nondense breasts in postmenopausal women. Premenopausal women have inherently increased breast density as compared with postmenopausal women per se. Previous data demonstrate that premenopausal women exhibit increased ADC compared with postmenopausal women [19, 38]. Even though these previous studies lack an accurate MRI-based breast density measure, their results are in line with our present finding of increased ADC in dense breasts [19, 38]. As malignant lesions may exhibit lower ADCs than benign lesions, the application of a specific threshold ADC may reduce biopsy rates without affecting sensitivity [18]. A background ADC dependent on breast density, however, may affect any thresholds decisive of biopsy. An ADC threshold of 1.6 × 10^−3^ mm^2^/s has been suggested as optimal for all lesion subtypes. How this threshold applies to normal breast tissue with varying density, however, is unknown. We show here that the ADC in nonmalignant tissue in nondense breasts falls below this threshold. Thus, we speculate that a low ADC per se may decrease the sensitivity of tumour detection. Whether ADC can be used as a sensitive distinguishing factor between malignant and benign lesions in nondense breast tissue remains to be elucidated. In addition, a condition that may affect the accuracy of ADC measurements is the choice of fat suppression, as that will influence SNR of the water resonance in nondense areas of the tissue [39, 40]; in this work, we have used SPAIR.

Our data also reveal that perfusion characteristics were significantly different between dense and nondense breast tissue, with delayed contrast dynamics in dense breasts. This may be explained by increased tissue pressure in dense breasts that could induce mechanical compression of blood vessels, resulting in changes in blood flow. Another potential reason is that fibroglandular tissues in general may have a delayed enhancement compared to blood vessels (or malignant highly perfused tumour lesions), resulting in a more delayed perfusion curve.

However, biological data suggest that dense breasts contain increased levels of inflammatory and angiogenic proteins that also affect the leakiness of vessels, resulting in partially dysfunctional vessels in the tissue.

Although breast tissue with varying density exhibits distinct differences on mammograms, very little is known about the underlying biological differences between dense and nondense breasts. Here, we performed comprehensive molecular characterisation of the extracellular microenvironment and tissue microstructure in breast tissue with varying densities. Our data suggest that there is a major biological difference between dense and nondense breasts, with a distinct profile of proteins related to inflammation, angiogenesis, and growth factors and/or metabolism in dense breasts. All these events are included among the hallmarks of cancer and represent a pro-tumorigenic microenvironment, which may enhance the ability of atypical cells to invade and progress into clinically definite cancer.

The present data show that potent inflammatory proteins correlate significantly with ADC. Corroborating our data from normal breast tissue without lesions, previous studies show that inflammatory lesions as determined by histology in tissues including the breast exhibit increased ADC [41, 42]. Inflammation and angiogenesis are closely intertwined processes, with many joint pathways. Inflammation leads to increased vessel permeability, which increases the number of inflammatory cells in the tissue that contribute to the release and production of angiogenic proteins. As we show here, many key regulators of these processes, including CCLs, CXCLs, soluble CD40, and AXIN1, were upregulated in dense breast tissue. This may lead to increased leakiness of blood vessels and oedema in the tissue and could explain the delayed peak of the contrast agent due to nonfunctional vessels and increased ADC.

Surprisingly, our data reveal increased levels of growth factors in dense breasts, including GH and FGF-19, which were significantly correlated with LTF, ADC, and perfusion tau. However, the mechanisms by which these proteins affect ADC and perfusion dynamics remain to be elucidated.

There are a few limitations of this work. One is the intermediately small number of subjects, on the other hand this was to some degree offset by the large number of measured parameters, and the combination of multimodal imaging and proteomics-based biomarkers. Another limitation of the MR measurements was that the SNR of the diffusion measurements potentially was biased by the varying degree of partial volume effects in individual imaging voxels. As the data were acquired using fat suppression, the remaining signal of the combined resonances was reduced, thus leading to lower relative SNR. Such intravoxel partial volume averaging effects potentially leading to a bias in the ADC of water has also been suggested elsewhere [20, 40]. Although not necessarily a limitation, the ADC-values are also to some degree affected by the choice of the largest b-values (in this work, we used up to 800 s/mm^2^) and may be a non-linear consequence of microstructural compartmentalisation. With respect to the perfusion measurements, blood vessels were not explicitly excluded, at least in most cases, as a priority was to place the VOIs in the regions of the upper left quadrant where the microdialysis was performed. Nevertheless, blood vessels should be apparent as hyperintense immediately after contrast injection, although this was not considered. Moreover, no 3D image registration was performed as we did not observe any visual indications of major registration differences between the scans.

In conclusion, normal breast tissue without lesions exhibits highly distinct patterns of ADC and perfusion characteristics depending on breast density. This may affect the sensitivity of these MRI-based parameters to identify aberrant lesions. In addition, we show that dense and nondense breast tissue are two essentially different biological entities with different environmental conditions that may promote invasive cancer development in dense breasts. This comprehensive characterisation of dense breast tissue reveals several novel pathways that may be further explored for the prevention of breast cancer in postmenopausal women with dense breasts. Such strategies may include therapeutics targeting inflammatory proteins, e.g., non-steroidal anti-inflammatory drugs, as well as therapies aiming at the inhibition of growth factor/metabolic pathways.

## Supplementary information

aj-checklist

Supplementary data

Supplementary Table 2

## Data Availability

Data are available upon reasonable request.
