# Supplementary material for: Breast density is strongly associated with multiparametric magnetic resonance imaging biomarkers and pro-tumorigenic proteins in situ
Source: Br J Cancer. 2022 Sep 22;127(11):2025–33. doi: 10.1038/s41416-022-01976-3 (PMC9681775; doi:10.1038/s41416-022-01976-3)
Supplement: Supplementary file 2 — Supplementary data [file 41416_2022_1976_MOESM2_ESM.pdf]

Supplement 1

STROBE diagram of the recruitment procedure.

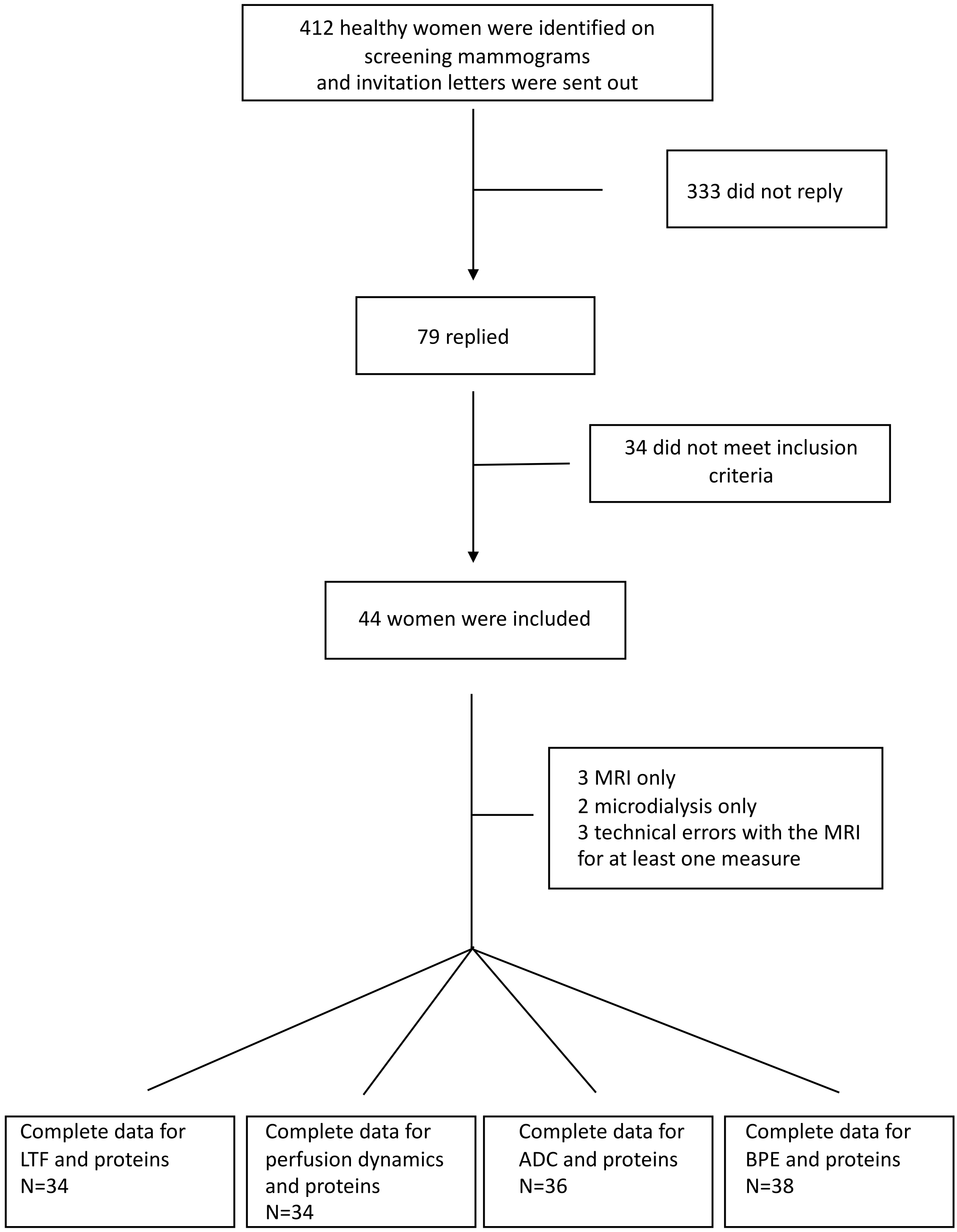

Breast Tissue Microstructure and Interpretation of Diffusion Data

The multimodal MR-measurements performed here, including diffusion, perfusion and LTF, may make it feasible to characterize the breast microstructure at a subvoxel level in an informed manner. In this section the details of these imaging-based investigations of the work are described in more detail.

In Fig. S1, the principal major structural elements are drawn inside an arbitrary sixed VOI (corresponding to an MRS-VOI) of the tissue; these include for simplicity lipid storage tissue (t1), stroma (t2) and epithelial tissue (t3). Each of these local environments may be further characterized by unique distributions of diffusion constants. An arbitrary image voxel contains typically, at least to some fraction, all of these different tissue types. Moreover, a VOI may contain a relatively large number of image voxels that show a distribution that depends on the tissue composition in each image voxel. I Fig. S1 three such diffusions distributions (ADC) are shown. The upper limit of diffusion is self-diffusing water molecules at the same temperature, characterized by a large $D_{free,water}$(ADC)-value (3.04E-3 mm^2/s; see https://dtrx.de/od/diff/). The histograms show the distributions of ADC in different tissue environments, ranging from non-dense breast tissue containing almost exclusively fat, to heterogeneous tissue with a mixture of fat and stroma (and some epithelial cells). These correspond to water *self-diffusion apparent temperatures* of -40 °C (in fat tissue) to 22 °C (in stroma), suggestive of the structurally restrictive nature of the fat tissue fraction, compared to stroma.

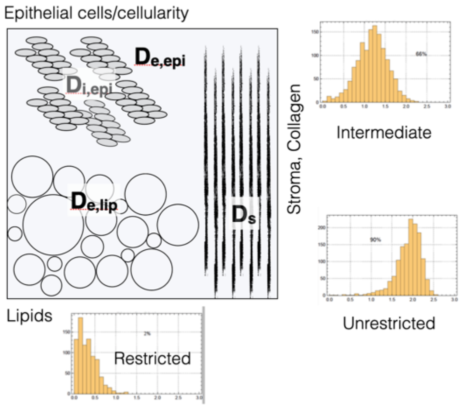

Fig. S1 Schematic illustration of different degrees of restricted diffusion (ADC) in breast tissue. *Breast tissue can schematically be described by regions containing different microscopic structures, each characterized by different apparent diffusion environments. The major microenvironments are the stromal environment containing collagen structures, and the lipid environment which may contain a large fraction of lipids (0-100%). Each of these are characterized by apparent diffusion coefficients* D_s *and* $D_{e,lip}$*, respectively. A smaller fraction of the tissue is epithelial cells (a few %), which are characterized by intracellular and extracellular apparent diffusion coefficients (*$D_{i,epi}$*, and* $D_{e,\ epi}$*, respectively).*

Consider that the ROI sample contains a few hundred image voxels, each containing a different mix of subvoxel volumes of different tissue types. The distribution of diffusion (ADC) values then will appear as separate distributions representative of different tissue types (left panel in Fig. S2), each which is restricting free water diffusion to a different extent. Or alternatively and more typically as a merged distribution, characterized by its width as well as central position. The width indicates the extent of microstructural heterogeneity (right panel in Fig. 2). Thus, larger IQR also suggests a larger microstructural subvoxel heterogeneity of the tissue.

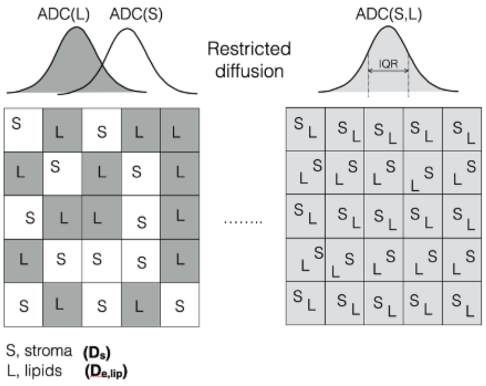

Fig. S2 Restricted diffusion (ADC) at the localized spatial level of breast tissue. *Assume that the tissue can be described by two separate major histological tissue types, stroma (S; including stromal collagen) and fat (L). And that the VOI used for tissue selection includes a large number of imaging voxels, as is schematically illustrated above. If the average microscopic structure level of the tissue is larger than, or similar to, the size of the image voxels, then a dual only partially overlapping ADC-distribution will be observed. In contrast, if the microscopic tissue distribution of fat and water is smaller than the image voxels, then a single distribution will be observed. The width of the predicted distribution as described by interquartile range (IQR) then reflects the sub-image-voxel extent of tissue heterogeneity.*

Diffusion Measurements and Microstructure

The cohort examined here was recruited as being dense and non-dense based on mammography radiological review, prior to their MR-examinations. The data was thus divided into these two groups for a group level analysis of the MR-data, and the results are shown in Fig. S3. Both left and right breast tissues were characterized by much less restricted molecular mobility in dense, as compared to non-dense tissue. Maximum as well as median ADC showed the same statistical appearance. Other measures such as IQR, Kurtosis and excess Kurtosis are often interpreted in terms of microstructural heterogeneity, and microstructural restriction respectively. As expected, the heterogeneity was larger in dense

breast tissue, but the spatial restraints were both more significant, and less variable, in nondense breast tissues.

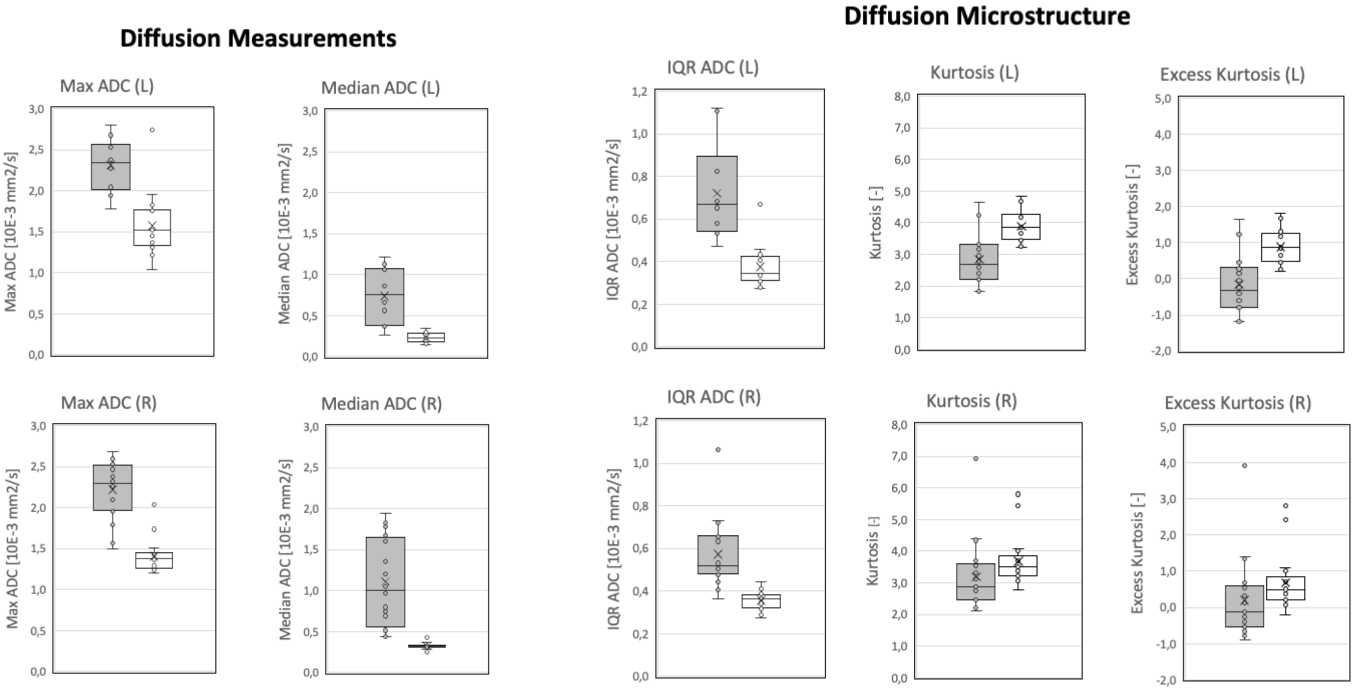

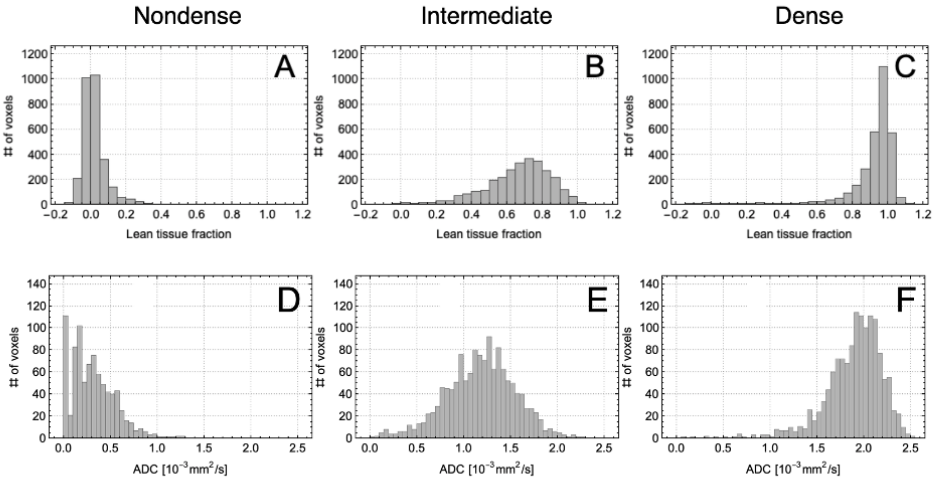

Fig. S3 Localized ADC in healthy dense (grey) and nondense (white) breasts. *(Top panels) Median, max and min, as well as excess Kurtosis (defined as Kurtosis - 3.0) and IQR of ADC, in the selected ROIs of right and left breasts, respectively, in units of* 10^{-3} *mm*^{2}*/s.*

LTF- and ADC-histograms of localized healthy nondense, intermediate and dense breast tissue. *(Bottom panels) (A–C) Lean tissue fraction, LTF, as well as ADC distributions are shown. Histograms of the standard ROIs for three different breast phenotypes (three different subjects) in the study, ranged from non-dense (A and D), through intermediary (B and E), to dense breast (F and C).*

Measurements of Lean Tissue Fraction

Similarly, the data obtained from LTF-measurements were significantly different, although a wide variation was quite evident, in spite of the relatively strict mammography based inclusion criteria that were used. The results of these measurements are shown in Fig. S4, a much larger variation was observed in dense tissue, than in non-dense.

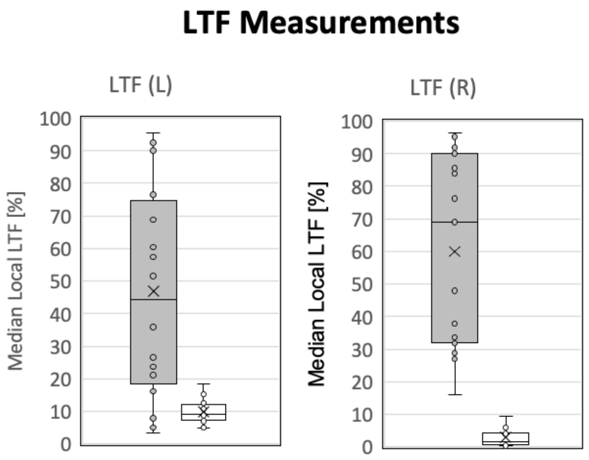

Fig. S4 Localized LTF in healthy dense (grey) and nondense (white) breasts, median and IQR. *The median LTF-value in non-dense breast in the selected ROIs of left and right breasts were significantly different from that in dense breasts. Moreover, the tissue heterogeneity as measured by LTF, was much larger in dense than in non-dense breast tissue.*

Perfusion Measurements and Microstructure

Perfusion showed a different aspect of the tissues, that is a reflection of capillary bed, as well as the potential different permeability of the blood vessels and extent of extracellular-extravascular space. The results from the perfusion experiments are shown in Fig. S4. A rapid entry of contrast agent in the tissue (*i.e.*, a short time-constant tau), corresponded to a larger total amount of contrast agent (as reflected by a larger AUC in the measurement interval).

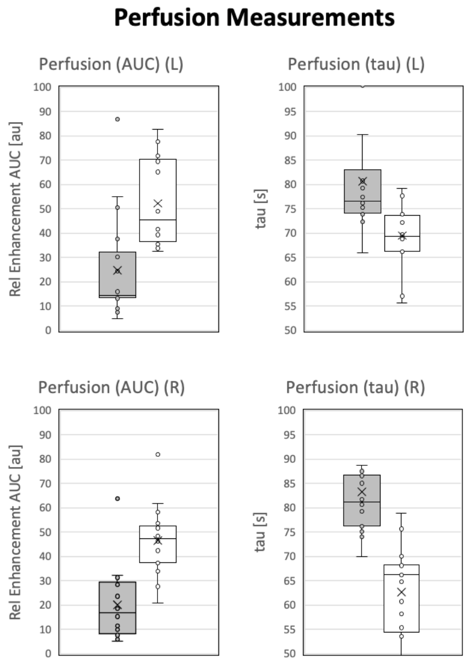

Fig. S5 Localized phenomenological perfusion parameters in healthy dense (grey) and nondense (white) breasts. *Time to peak (tau, in s), and the area under the perfusion curve (AUC) minus the baseline, up until 6 min of perfusion.*

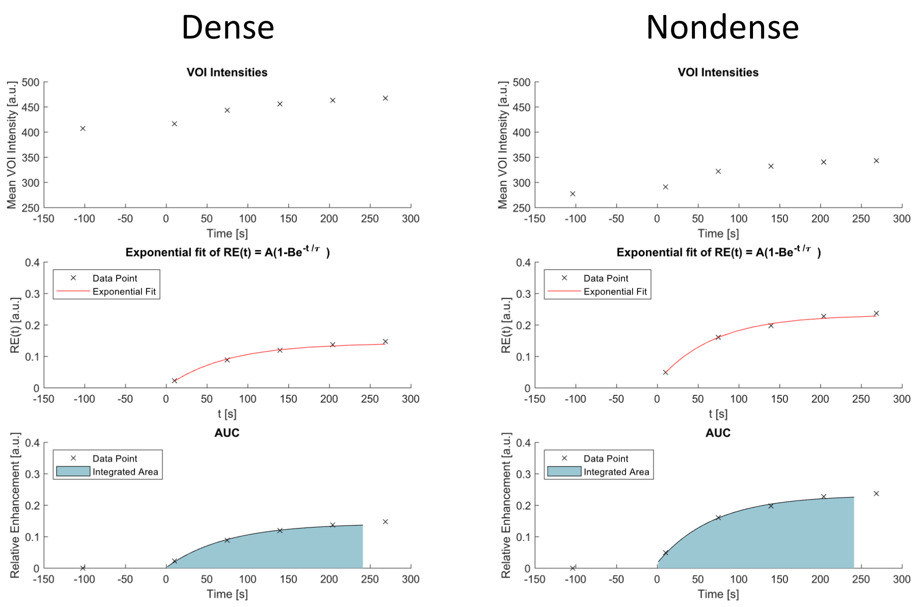

Fig. S6 Typical examples of the MR-perfusion curves in healthy dense (left) and nondense (right) breasts (all these were obtained from left breasts). *(TOP) experimental data, (MIDDLE) Exponential fit, time to peak (tau, in s) was determined using an exponential fit, and (BOTTOM) the area under the perfusion curve (AUC) (i.e., 'relative enhancement') is shown in blue, up until 6 min of perfusion.*

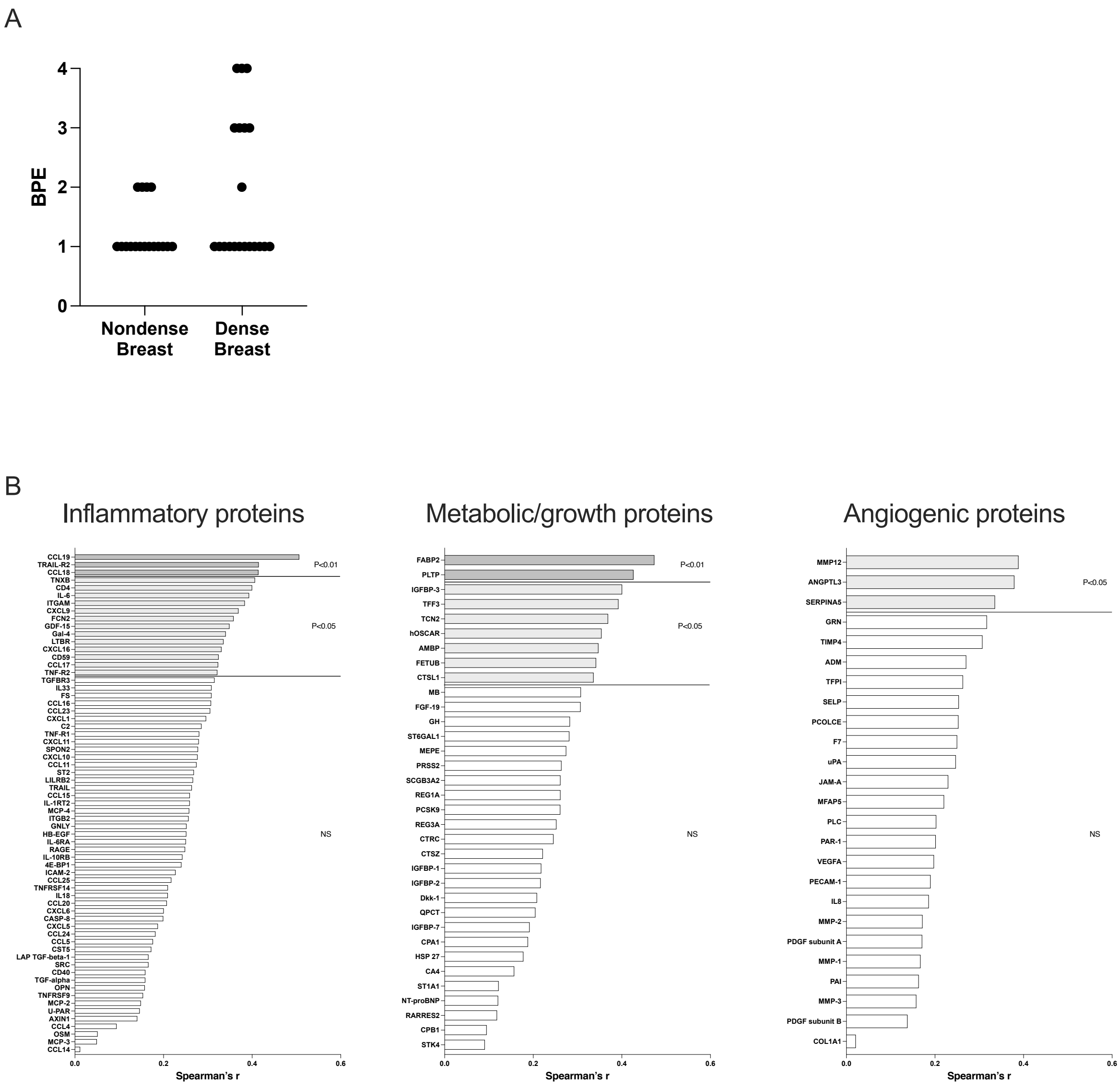

Fig. S7 BPE - Background parenchymal enhancement.

A. Categories in nondense and dense breasts. B. Correlations between BPE and proteins of different categories.

Summary of MR-Measurements

Finally, a tabular description of the MR-measurements (in the *left* breast; with a ROI located in the same area where the microdialysis was performed, subsequent to the MR-measurements) is shown in Table S1. Differences were very significant with respect to all MR-measures, between the two groups.

Table S1. *Summary, nondense vs. dense, localized ADC, LTF and perfusion parameters in healthy tissue, results from measurements of left breast tissues. For measurements of right breast tissue, see diagrams above. Classification into the two categories nondense and dense is based on mammography review by an experienced radiologist. However, the circumstances of the MR-measurements were different, which resulted in some overlap between the categories.*

	**Nondense**	**Dense**
**ADC** (10^{-3} mm^2/s)		
n	17	20
Average Mean±SD (p<0.001)	**0.27**±0.25	**0.75**±0.46
Median (p<0.001)	**0.20**	**0.65**
Median IQR (p<0.001)	**0.32**	**0.67**
Median [max]	[0.31]	[1.52]
Median Exc Kurtosis (p<0.001)	**0.86**	**-0.32**
**LTF** (fraction)		
n	19	18
Average mean±SD (p<0.001)	**0.04**±0.04	**0.47**±0.23
Median (p<0.001)	0.01	0.44
Median [min; max]	[0.00; 0.03]	[0.04; 0.95]
**Perfusion, tau (s)**		
n	16	18
Average tau±SD (p<0.01)	**69.5**±6.7	**80.6**±10.8
Median tau	69.3	76.6
**Perfusion, AUC (AU)**		
n	16	18
Average AUC±SD (p<0.001)	**52.2**±17.5	**24.7**±14.4
Median AUC	45.3	14.4
