# Supplementary material for: Breast density is strongly associated with multiparametric magnetic resonance imaging biomarkers and pro-tumorigenic proteins in situ
Source: Br J Cancer. 2022 Sep 22;127(11):2025–33. doi: 10.1038/s41416-022-01976-3 (PMC9681775; doi:10.1038/s41416-022-01976-3)
Supplement: Supplementary file 3 — Supplementary Table 2 [file 41416_2022_1976_MOESM3_ESM.pdf]

Analyzed protein	D=detecable (above Limit of Detection (LOD) in >50% the samples) and ND=non-detecable
2,4-dienoyl-CoA reductase, mitochondrial (DECR1)	ND
A disintegrin and metalloproteinase with thrombospondin motifs 13 (ADAM-TS13)	ND
Adenosine Deaminase (ADA)	D
ADM (ADM)	D
Agouti-related protein (AGRP)	ND
Alpha-L-iduronidase (IDUA)	ND
Aminopeptidase N (AP-N)	D
Angiogenin (ANG)	D
Angiopoietin-1 (ANGPT1)	ND
Angiopoietin-1 receptor (TIE2)	ND
Angiopoietin-related protein 3 (ANGPTL3)	D
Angiotensin-converting enzyme 2 (ACE2)	ND
Apolipoprotein M (APOM)	D
Artemin (ARTN)	ND
Axin-1 (AXIN1)	D
Azurocidin (AZU1)	D
Beta-Ala-His dipeptidase (CNDP1)	D
Beta-galactoside alpha-2,6-sialyltransferase 1 (ST6GAL1)	D
Beta-nerve growth factor (Beta-NGF)	ND
Bleomycin hydrolase (BLM hydrolase)	ND
Bone morphogenetic protein 6 (BMP-6)	ND
Brother of CDO (BOC)	ND
C-C motif chemokine 14 (CCL14)	D
C-C motif chemokine 15 (CCL15)	D
C-C motif chemokine 16 (CCL16)	D
C-C motif chemokine 17 (CCL17)	D
C-C motif chemokine 18 (CCL18)	D
C-C motif chemokine 19 (CCL19)	D

C-C motif chemokine 20 (CCL20)	D
C-C motif chemokine 23 (CCL23)	D
C-C motif chemokine 24 (CCL24)	D
C-C motif chemokine 25 (CCL25)	D
C-C motif chemokine 28 (CCL28)	D
C-C motif chemokine 3 (CCL3)	D
C-C motif chemokine 4 (CCL4)	D
C-C motif chemokine 5 (CCL5)	D
C-X-C motif chemokine 1 (CXCL1)	D
C-X-C motif chemokine 10 (CXCL10)	D
C-X-C motif chemokine 11 (CXCL11)	D
C-X-C motif chemokine 16 (CXCL16)	D
C-X-C motif chemokine 5 (CXCL5)	D
C-X-C motif chemokine 6 (CXCL6)	D
C-X-C motif chemokine 9 (CXCL9)	D
Cadherin-1 (CDH1)	D
Cadherin-5 (CDH5)	ND
Carbonic anhydrase 1 (CA1)	D
Carbonic anhydrase 3 (CA3)	D
Carbonic anhydrase 4 (CA4)	D
Carbonic anhydrase 5A, mitochondrial (CA5A)	ND
Carboxypeptidase A1 (CPA1)	D
Carboxypeptidase B (CPB1)	D
Carcinoembryonic antigenrelated cell adhesion molecule 8 (CEACAM8)	D
Cartilage acidic protein 1 (CRTAC1)	D
Cartilage oligomeric matrix protein (COMP)	D
Caspase 8 (CASP-8)	D
Caspase-3 (CASP-3)	D
Cathepsin D (CTSD)	D
Cathepsin L1 (CTSL1)	D
Cathepsin Z (CTSZ)	D
CD166 antigen (ALCAM)	D
CD40 ligand (CD40-L)	D

CD40L receptor (CD40)	D
CD59 glycoprotein (CD59)	D
Chitinase-3-like protein 1 (CHI3L1)	D
Chitotriosidase-1 (CHIT1)	D
Chymotrypsin C (CTRC)	D
Coagulation factor VII (F7)	D
Coagulation factor XI (F11)	D
Collagen alpha-1(I) chain (COL1A1)	D
Collagen alpha-1(XVIII) chain (COL18A1)	D
Complement C1q tumor necrosis factor-related protein 1 (C1QTNF1)	D
Complement C2 (C2)	D
Complement component C1q receptor (CD93)	D
Complement factor H-related protein 5 (CFHR5)	D
Complement receptor type 2 (CR2)	D
Contactin-1 (CNTN1)	D
CUB domain-containing protein 1 (CDCP1)	ND
Cystatin D (CST5)	D
Cystatin-B (CSTB)	D
Cystatin-C (CST3)	D
Decorin (DCN)	D
Delta and Notch-like epidermal growth factor- related recep (DNER)	ND
Dickkopf-related protein 1 (Dkk-1)	D
Dipeptidyl peptidase 4 (DPP4)	D
E-selectin (SELE)	ND
EGF-containing fibulin-like extracellular matrix protein 1 (EFEMP1)	D
Elafin (PI3)	D
Endoglin (ENG)	D
Eotaxin-1 (CCL11)	D
Ephrin type-B receptor 4 (EPHB4)	D
Epidermal growth factor receptor (EGFR)	D
Epithelial cell adhesion molecule (Ep-CAM)	D
Eukaryotic translation initiation factor 4E-binding protein 1 (4E-BP1)	D
Fatty acid-binding protein, adipocyte (FABP4)	D

Fatty acid-binding protein, intestinal (FABP2)	D
Fetuin-B (FETUB)	D
Fibroblast growth factor 19 (FGF-19)	D
Fibroblast growth factor 21 (FGF-21)	ND
Fibroblast growth factor 23 (FGF-23)	ND
Fibroblast growth factor 5 (FGF-5)	ND
Ficolin-2 (FCN2)	D
Fms-related tyrosine kinase 3 ligand (Flt3L)	D
Follistatin (FS)	D
Fractalkine (CX3CL1)	D
Galectin-3 (Gal-3)	D
Galectin-4 (Gal-4)	D
Galectin-9 (Gal-9)	D
Gastric intrinsic factor (GIF)	D
Gastrotropin (GT)	ND
Glial cell line-derived neurotrophic factor (GDNF)	ND
Glutaminyl-peptide cyclotransferase (QPCT)	D
Granulins (GRN)	D
Granulysin (GNLY)	D
Growth arrest-specific protein 6 (GAS6)	D
Growth hormone (GH)	D
Growth/differentiation factor 15 (GDF-15)	D
Growth/differentiation factor 2 (GDF-2)	ND
Heat shock 27 kDa protein (HSP 27)	D
Heme oxygenase 1 (HO-1)	D
Hepatocyte growth factor (HGF)	D
Hepatocyte growth factor receptor (MET)	D
Hydroxyacid oxidase 1 (HAOX1)	ND
Ig lambda-2 chain C regions (IGLC2)	D
Insulin-like growth factor-binding protein 1 (IGFBP-1)	D
Insulin-like Growth Factor-Binding Protein 2 (IGFBP-2)	D
Insulin-like growth factor-binding protein 3 (IGFBP3)	D
Insulin-like growth factor-binding protein 6 (IGFBP6)	D

Insulin-like growth factor-binding protein 7 (IGFBP-7)	D
Integrin alpha-M (ITGAM)	D
Integrin beta-2 (ITGB2)	D
Intercellular adhesion molecule 1 (ICAM1)	D
Intercellular adhesion molecule 2 (ICAM-2)	D
Intercellular adhesion molecule 3 (ICAM3)	D
Interferon gamma (IFN-gamma)	ND
Interleukin-1 alpha (IL-1 alpha)	D
Interleukin-1 receptor antagonist protein (IL-1ra)	D
Interleukin-1 receptor type 1 (IL-1RT1)	D
Interleukin-1 receptor type 2 (IL-1RT2)	D
Interleukin-10 (IL-10)	ND
Interleukin-10 receptor subunit alpha (IL-10RA)	ND
Interleukin-10 receptor subunit beta (IL-10RB)	D
Interleukin-12 subunit beta (IL-12B)	ND
Interleukin-13 (IL-13)	ND
Interleukin-15 receptor subunit alpha (IL-15RA)	ND
Interleukin-17 receptor A (IL-17RA)	D
Interleukin-17A (IL-17A)	ND
Interleukin-17C (IL-17C)	ND
Interleukin-17D (IL-17D)	ND
Interleukin-18 (IL-18)	D
Interleukin-18 receptor 1 (IL-18R1)	D
Interleukin-18-binding protein (IL-18BP)	D
Interleukin-2 (IL-2)	ND
Interleukin-2 receptor subunit alpha (IL2-RA)	D
Interleukin-2 receptor subunit beta (IL-2RB)	ND
Interleukin-20 (IL-20)	ND
Interleukin-20 receptor subunit alpha (IL-20RA)	ND
Interleukin-22 receptor subunit alpha-1 (IL-22 RA1)	ND
Interleukin-24 (IL-24)	ND
Interleukin-27 (IL-27)	ND
Interleukin-33 (IL-33)	D

Interleukin-4 (IL-4)	ND
Interleukin-4 receptor subunit alpha (IL-4RA)	ND
Interleukin-5 (IL-5)	ND
Interleukin-6 (IL-6)	D
Interleukin-6 receptor subunit alpha (IL-6RA)	D
Interleukin-7 (IL-7)	ND
Interleukin-7 receptor subunit alpha (IL7R)	D
Interleukin-8 (IL-8)	D
Junctional adhesion molecule A (JAM-A)	D
Kallikrein-6 (KLK6)	D
Kidney injury molecule 1 (KIM1)	ND
L-selectin (SELL)	D
Lactoylglutathione lyase (GLO1)	D
Latency-associated peptide transforming growth factor beta 1 (LAP TGF-beta-1)	D
Latent-transforming growth factor beta-binding protein 2 (LTBP2)	D
Lectin-like oxidized LDL receptor 1 (LOX-1)	D
Leptin (LEP)	D
Leukemia inhibitory factor (LIF)	D
Leukemia inhibitory factor receptor (LIF-R)	ND
Leukocyte immunoglobulin-like receptor subfamily B member 1 (LILRB1)	D
Leukocyte immunoglobulin-like receptor subfamily B member 2 (LILRB2)	D
Leukocyte immunoglobulin-like receptor subfamily B member 5 (LILRB5)	D
Lipoprotein lipase (LPL)	D
Lithostathine-1-alpha (REG1A)	D
Liver carboxylesterase 1 (CES1)	D
Low affinity immunoglobulin gamma Fc region receptor II-a (FCGR2A)	D
Low affinity immunoglobulin gamma Fc region receptor II-b (IgG Fc receptor II-b)	ND
Low affinity immunoglobulin gamma Fc region receptor III-B (FCGR3B)	D
Low-density lipoprotein receptor (LDL receptor)	D
Lymphatic vessel endothelial hyaluronic acid receptor 1 (LYVE1)	D
Lymphotactin (XCL1)	ND
Lymphotoxin-beta receptor (LTBR)	D
Lysosomal Pro-X carboxypeptidase (PRCP)	D

Macrophage colony-stimulating factor 1 (CSF-1)	D
Macrophage receptor MARCO (MARCO)	ND
Mannose-binding protein C (MBL2)	D
Mast/stem cell growth factor receptor Kit (KIT)	D
Matrix extracellular phosphoglycoprotein (MEPE)	D
Matrix metalloproteinase-1 (MMP-1)	D
Matrix metalloproteinase-10 (MMP-10)	D
Matrix metalloproteinase-12 (MMP12)	D
Matrix metalloproteinase-2 (MMP-2)	D
Matrix metalloproteinase-3 (MMP-3)	D
Matrix metalloproteinase-7 (MMP7)	D
Matrix metalloproteinase-9 (MMP-9)	D
Melusin (ITGB1BP2)	ND
Membrane cofactor protein (CD46)	D
Membrane primary amine oxidase (AOC3)	D
Metalloproteinase inhibitor 1 (TIMP1)	D
Metalloproteinase inhibitor 4 (TIMP4)	D
Microfibrillar-associated protein 5 (MFAP5)	D
Monocyte chemotactic protein 1 (MCP-1) CCL2	D
Monocyte chemotactic protein 2 (MCP-2) CCL8	D
Monocyte chemotactic protein 3 (MCP-3) CCL7	D
Monocyte chemotactic protein 4 (MCP-4) CCL13	D
Multiple epidermal growth factor-like domains protein 9 (MEGF9)	D
Myeloblastin (PRTN3)	D
Myeloperoxidase (MPO)	D
Myoglobin (MB)	D
N-terminal prohormone brain natriuretic peptide (NT-proBNP)	D
Natriuretic peptides B (BNP)	ND
Natural killer cell receptor 2B4 (CD244)	ND
Neural cell adhesion molecule 1 (NCAM1)	D
Neural cell adhesion molecule L1-like protein (CHL1)	D
Neurogenic locus notch homolog protein 1 (NOTCH1)	D
Neurogenic locus notch homolog protein 3 (Notch 3)	ND

Neuropilin-1 (NRP1)	D
Neurotrophin-3 (NT-3)	ND
Neurturin (NRTN)	ND
Neutrophil defensin 1 (DEFA1)	D
Neutrophil gelatinase-associated lipocalin (LCN2)	D
NF-kappa-B essential modulator (NEMO)	D
Nidogen-1 (NID1)	D
Oncostatin-M (OSM)	D
Oncostatin-M-specific receptor subunit beta (OSMR)	D
Osteoclast-associated immunoglobulin- like receptor (hOSCAR)	D
Osteopontin (OPN)	D
Osteoprotegerin (OPG)	D
P-selectin (SELP)	D
P-selectin glycoprotein ligand 1 (PSGL-1)	ND
Pappalysin-1 (PAPPA)	ND
Paraoxonase (PON 3)	D
Pentraxin-related protein PTX3 (PTX3)	ND
Peptidoglycan recognition protein 1 (PGLYRP1)	D
Peptidyl-glycine alpha-amidating monooxygenase (PAM)	D
Perlecan (PLC)	D
Phospholipid transfer protein (PLTP)	D
Placenta growth factor (PGF)	D
Plasma serine protease inhibitor (SERPINA5)	D
Plasminogen activator inhibitor 1 (PAI)	D
Platelet endothelial cell adhesion molecule (PECAM-1)	D
Platelet glycoprotein Ib alpha chain (GP1BA)	D
Platelet glycoprotein VI (GP6)	ND
Platelet-activating factor acetylhydrolase (PLA2G7)	ND
Platelet-derived growth factor subunit A (PDGF subunit A)	D
Platelet-derived growth factor subunit B (PDGF subunit B)	D
Plexin-B2 (PLXNB2)	D
Poly [ADP-ribose] polymerase 1 (PARP-1)	D
Polymeric immunoglobulin receptor (PIgR)	D

Pro-interleukin-16 (IL16)	D
Procollagen C-endopeptidase enhancer 1 (PCOLCE)	D
Programmed cell death 1 ligand 1 (PD-L1)	ND
Programmed cell death 1 ligand 2 (PD-L2)	ND
Proheparin-binding EGF-like growth factor (HB-EGF)	D
Prolargin (PRELP)	D
Prolyl endopeptidase FAP (FAP)	D
Proprotein convertase subtilisin/kexin type 9 (PCSK9)	D
Prostasin (PRSS8)	D
Protein AMBP (AMBP)	D
Protein delta homolog 1 (DLK-1)	D
Protein S100-A12 (EN-RAGE)	D
Protein-glutamine gamma- glutamyltransferase 2 (TGM2)	D
Proteinase-activated receptor 1 (PAR-1)	D
Proto-oncogene tyrosine-protein kinase Src (SRC)	D
Pulmonary surfactant-associated protein D (PSP-D)	ND
Receptor for advanced glycosylation end products (RAGE)	D
Receptor-type tyrosine-protein phosphatase S (PTPRS)	D
Regenerating islet-derived protein 3-alpha (REG3A)	D
Renin (REN)	ND
Resistin (RETN)	D
Retinoic acid receptor responder protein 2 (RARRES2)	D
Scavenger receptor cysteine-rich type 1 protein M130 (CD163)	ND
Secretoglobin family 3A member 2 (SCGB3A2)	D
Serine protease 27 (PRSS27)	D
Serine/threonine-protein kinase 4 (STK4)	D
Serpin A12 (SERPINA12)	ND
Serum amyloid A-4 protein (SAA4)	D
Signaling lymphocytic activation molecule (SLAMF1)	ND
SIR2-like protein 2 (SIRT2)	ND
SLAM family member 5 (CD84)	ND
SLAM family member 7 (SLAMF7)	ND
Sortilin (SORT1)	ND

SPARC-like protein 1 (SPARCL1)	D
Spondin-1 (SPON1)	D
Spondin-2 (SPON2)	D
ST2 protein (ST2)	D
STAM-binding protein (STAMPB)	D
Stem cell factor (SCF)	D
Sulfotransferase 1A1 (ST1A1)	D
Superoxide dismutase [Cu-Zn] (SOD1)	D
Superoxide dismutase [Mn], mitochondrial (SOD2)	D
T-cell immunoglobulin and mucin domain-containing protein 4 (TIMD4)	D
T-cell surface glycoprotein CD4 (CD4)	D
T-cell surface glycoprotein CD5 (CD5)	ND
T-cell surface glycoprotein CD6 isoform (CD6)	ND
T-cell surface glycoprotein CD8 alpha chain (CD8A)	ND
Tartrate-resistant acid phosphatase type 5 (TR-AP)	D
Tenascin (TNC)	ND
Tenascin-X (TNXB)	D
Thrombomodulin (TM)	ND
Thrombopoietin (THPO)	ND
Thrombospondin-2 (THBS2)	ND
Thrombospondin-4 (THBS4)	D
Thymic stromal lymphopoietin (TSLP)	ND
Thyroxine-binding globulin (SERPINA7)	D
Tissue factor (TF)	D
Tissue factor pathway inhibitor (TFPI)	D
Tissue-type plasminogen activator (t-PA)	D
TNF-beta (TNFB)	ND
TNF-related activation-induced cytokine (TRANCE)	ND
TNF-related apoptosis-inducing ligand (TRAIL)	D
TNF-related apoptosis-inducing ligand receptor 2 (TRAIL-R2)	D
Transcobalamin-2 (TCN2)	ND
Transferrin receptor protein 1 (TR)	D
Transforming growth factor alpha (TGF-alpha)	D

Transforming growth factor beta receptor type 3 (TGFBR3)	D
Transforming growth factor-beta-induced protein ig-h3 (TGFBI)	D
Trefoil factor 3 (TFF3)	D
Trem-like transcript 2 protein (TLT-2)	D
Trypsin-2 (PRSS2)	D
Tumor necrosis factor (Ligand) superfamily, member 12 (TWEAK)	D
Tumor necrosis factor (TNF)	ND
Tumor necrosis factor ligand superfamily member 13B (TNFSF13B)	D
Tumor necrosis factor ligand superfamily member 14 (TNFSF14)	D
Tumor necrosis factor receptor 1 (TNF-R1)	D
Tumor necrosis factor receptor 2 (TNF-R2)	D
Tumor necrosis factor receptor superfamily member 10A (TNFRSF10A)	ND
Tumor necrosis factor receptor superfamily member 10C (TNFRSF10C)	D
Tumor necrosis factor receptor superfamily member 11A (TNFRSF11A)	D
Tumor necrosis factor receptor superfamily member 13B (TNFRSF13B)	D
Tumor necrosis factor receptor superfamily member 14 (TNFRSF14)	D
Tumor necrosis factor receptor superfamily member 6 (FAS)	D
Tumor necrosis factor receptor superfamily member 9 (TNFRSF9)	D
Tyrosine-protein kinase Mer (MERTK)	ND
Tyrosine-protein kinase receptor Tie-1 (TIE1)	D
Tyrosine-protein kinase receptor UFO (AXL)	D
Tyrosine-protein phosphatase non- receptor type substrate 1 (SHPS-1)	D
Urokinase plasminogen activator surface receptor (U-PAR)	D
Urokinase-type plasminogen activator (uPA)	D
Uromodulin (UMOD)	ND
V-set and immunoglobulin domain- containing protein 2 (VSIG2)	ND
Vascular cell adhesion protein 1 (VCAM1)	D
Vascular Endothelial Growth factor A (VEGF-A)	D
Vascular endothelial growth factor D (VEGFD)	D
Vasorin (VASN)	D
Vitamin K-dependent protein C (PROC)	D
von Willebrand factor (vWF)	D
